# Novel Influenza H1N1 in Pregnancy: A Report of Two Cases

**DOI:** 10.1155/2009/514353

**Published:** 2010-01-28

**Authors:** Dmitry Fridman, Oumar Kuzbari, Howard Minkoff

**Affiliations:** Department of Obstetrics and Gynecology, Maimonides Medical Center, Brooklyn, NY 11219, USA

## Abstract

*Background*. Pregnant women are a high-risk group for morbidity and mortality from influenza. During the current pandemic of H1N1 influenza, few cases of H1N1 have been reported in pregnancy. *Cases*. We report two cases of H1N1 influenza which occurred in single institution in the course of one month. The first patient developed acute respiratory distress syndrome, required intubation, and eventually died. The second patient had influenza H1N1 that did not have any major sequela. *Conclusion*. H1N1 influenza in pregnancy can be associated with severe complications. Widespread vaccination, when available, prompt diagnosis, and adequate treatment with antiviral medications when infection occurs are required.

## 1. Introduction

During seasonal influenza epidemics, pregnant women constitute a high-risk group for disease-related morbidity and mortality [1]. During current infection pregnancy, seems to be a major risk factor of lethal outcome [2]. There are also reports of an increased risk of miscarriage, birth defect, and preterm delivery when pregnancy is associated with influenza infection [3, 4]. However, much less is known about pregnancy and novel influenza A (H1N1). 

The first lethal case of respiratory infection with H1N1 in an adult in the United States was diagnosed in a pregnant woman on April 30 [5, 6]. To our knowledge (after reviewing pubmed, google scholar, and cochrane data base), only two case series totaling eight patients of H1N1 in pregnancy have been reported (none of which appeared in the obstetrical literature), with all suggesting a complicated clinical course [4–6].

## 2. Cases

### 2.1. Patient A

On May 26, 2009, a 27-year old middle-eastern P0000 at 32 weeks of gestation with no significant past medical history presented complaining of cough with blood-tinged sputum and fevers for four days. The patient did not recall any contacts with ill individuals and denied recent travel. She had been evaluated in the emergency room two days prior to admission and had been diagnosed with viral syndrome which had not improved. Her vital signs were pulse 110, respiratory rate 22, blood pressure 119/74, and temperature 100.1 F, while her oxygen saturation ranged from 93% to 96%. On physical examination, she had bilateral ronchi in her lungs. Her white blood count (WBC) was 6.6 K/Ul. Her chest X-ray revealed bilateral middle and lower lobe infiltrates consistent with pneumonia. When the patient was admitted, she started on ceftriaxone and azithromycin. Her nasal swab was negative for influenzas both A and B, and when repeated three days later, it was again negative. 

The next day the patient developed acute respiratory distress syndrome, had difficulty breathing, had a respiratory rate of 22–28, and a temperature of 102.0 her pulse oxygen saturation declined to 86. Due to the deterioration of her respiratory functions, she was intubated and transferred to the ICU. She started on piperacillin-tazobactam and vancomycin, and on hospital day four, she started on oseltamivir. On hospital day five her condition continued to deteriorate, with her arterial oxygen saturation decreasing to 88% on 100% FiO_2_. The patient had previously stated that a cesarean delivery should be performed only if it carried no risk for her, and she named her husband as her health care proxy. When the ICU staff felt she could no longer be sustained even with hand bagging a decision to perform, a cesarean section was made in the hope that it would ameliorate the mother's condition. After the health care proxy agreed, a cesarean delivery was performed in the ICU and a female infant weighing 1500 g, with Apgar scores of 1 and 1, was delivered. The newborn died on day one with evidence of chronic hypoxia. In the postoperative period the patient's oxygenation status showed some improvement but she was still requiring high FiO_2_ to maintain proper oxygenation. Over the next days the patient's condition remained critical. On hospital day 12 the H1N1 influenza nasopharyngeal swab taken on day 3 was reported as positive by the New York Department of Health (DOH). Ultimately, 17 days after admission, due to her deteriorating oxygenation status, sepsis, and progressive ARDS, the decision was made to transfer the patient to another center for extracorporeal membrane oxygenation. However, the patient expired 25 days after transfer to that institution. Patients autopsy report was not available.

### 2.2. Patient B

The patient was a 29-year old white P1011 at 37 weeks of gestation who presented in early labor with fever, dry cough, headache, nausea, and vomiting. Her prenatal course was uneventful. She had a temperature of 102.3, a respiratory rate of 22, a heart rate of 105, and a pulse oxygen saturation of 100% in room air. She reported that her daughter had some respiratory symptoms and fever a few days earlier. Her lab results were unremarkable, with a white blood count of 4.9, with 8% lymphocytes. 

The patient was having irregular contractions, and her cervix was 1-2 cm open and 70% effaced. The fetal heart rate was 170 with moderate variability. Chorioamnionitis was suspected and she was admitted for labor augmentation. Because of the ongoing epidemic of H1N1 in the community, she was isolated with droplet precautions, placed in negative pressure room, and started on oseltamivir and ampicillin-clavulanic acid. The patient was augmented with oxytocin and had an uncomplicated vaginal delivery in 8 hours of a female infant weighing 3220 g, Apgar 9/9. She had an uneventful postpartum period and was discharged on postpartum day 2. Nasopharyngeal swab cultures that were sent on admission were reported as positive for influenzas A on the next day, and novel influenza H1N1 was confirmed by the DOH. The newborn tested negative for influenza A and B by real-time reverse transcription PCR. On her six-week postpartum visit she was asymptomatic, and the baby was doing well.

## 3. Discussion

This series of two patients with novel influenza A (H1N1) virus demonstrates the variation in course that the disease may take in pregnancy. The first case was a pregnant woman with late initiation of antiviral therapy. The patient rapidly progressed to ARDS despite the fact that all the supportive measures expired. This is illustrative of the importance of early initiation of antiviral therapy since delayed initiation has not been shown to have a salutary effect on individuals with H1N1. A second case—apparently a more frequent scenario with mild viral symptoms—was appropriately managed and had no major sequel. Though mild cases are undoubtedly underrepresented in our report (as they are in other ones), it is reasonable to suggest that, as with seasonal influenza, pregnant women constitute a population at risk for morbidity and mortality. 

Patients with H1N1 viral infection present with acute respiratory symptoms—dry cough, sore throat, nasal congestion, and fever. Almost one third of them report contact with an ill individual on admission [7]. The symptoms are nonspecific such that it is not surprising that many patients later confirmed to have swine flu (including those in our report), had had their symptoms attributed to the common cold when they had been seen earlier [5, 6]. The CDC recommends that clinicians use nasopharyngeal swabs for rapid detection of antigens for influenzas A and B in patients with fever and respiratory symptoms. If an unsubtypable influenza A virus infection is found, the specimen should be sent to a state public health laboratory for additional testing to identify H1N1 virus using the real-time PCR technique which is currently recommended for laboratory confirmation of H1N1 viral infection [8]. Antiviral therapy is often delayed for pregnant patients [4–7], as it was in our case, and it should be reinforced that antiviral therapy should be started as soon as possible based only on clinical presentation of fever and sore throat or cough without waiting to obtain results of laboratory testing, unless another cause of symptoms is reasonably suspected [9]. It is preferable to start antiviral therapy within 48 hours of the first influenza-related symptoms and continue for 5 days. H1N1 virus is sensitive to both oseltamivir (75 mg twice a day) and zanamivir (two 5 mg inhalations twice a day) [10]. Due to systemic absorption and more experience with oseltamivir, it is the preferred medication. The benefit of treating the influenza outweighs any theoretical risk for the fetus. In addition to an antiviral preparation, acetaminophen should also be started because fever may be associated with neural tube defect, neonatal seizures, encephalopathy, cerebral palsy, and neonatal death [3, 9]. It is recommended to treat severe cases of H1N1 infection in a hospital, using respiratory support with supplemental oxygen and mechanical ventilation as required. Antibiotic supplementation should be guided by the presence of pneumonia depending on the patterns of resistance in the region [11]. Since pneumococcal pneumonia is frequently a secondary invader, pregnant women who are in risk groups should also be vaccinated against that organism. 

Current CDC recommendation suggests that pregnant women who had contact with someone suspected of infection with novel H1N1 influenza virus should receive prophylactic treatment with either oseltamivir (75 mg daily) or zanamivir (two 5 mg inhalations daily) for 10 days. The CDC states that zanamivir is preferable due to its low systemic absorption, but because of its inhaled route of administration, respiratory complications must be considered [9]. H1N1 vaccine is planned to be available by mid-October, produced in a similar fashion as that of the seasonal vaccine. The advisory committee on immunization safety recommends that pregnant women be included in primary targeted groups for vaccination [12]. Vaccination is also recommended for household contacts of pregnant women; however, chemoprophylaxis is not indicated for otherwise healthy people exposed to influenza. Early empiric treatment should be started if symptoms arise [13]. 

Currently all subtyped influenza A viruses reported to CDC (99% of all specimens sent to CDC) are H1N1 [14]. Obstetricians should be prepared to diagnose and rapidly treat H1N1 and, now with the availability of H1N1 vaccine, to be proactive with vaccination programs to blunt the spread of the infection.

**Addendum**

Since this article was initially submitted, two additional patients with H1N1, including one who ended up on ECMO and the other in the ICU, have been cared for at our facility.
